# Lymphocytes from B-acute lymphoblastic leukemia patients present differential regulation of the adenosinergic axis depending on risk stratification

**DOI:** 10.1007/s12672-022-00602-1

**Published:** 2022-12-30

**Authors:** Vitória Brum da Silva Nunes, Camila Kehl Dias, Juliete Nathali Scholl, Alexia Nedel Sant’Ana, Amanda de Fraga Dias, Mariela Granero Farias, Ana Paula Alegretti, Monalisa Sosnoski, Liane Esteves Daudt, Mariana Bohns Michalowski, Ana Maria Oliveira Battastini, Alessandra Aparecida Paz, Fabrício Figueiró

**Affiliations:** 1 Laboratório de Imunobioquímica do Câncer, Departamento de Bioquímica, Instituto de Ciências Básicas da Saúde, UFRGS, Porto Alegre, RS CEP 90035-003 Brazil; 2 Programa de Pós-Graduação em Ciências Biológicas: Bioquímica, Instituto de Ciências Básicas da Saúde, UFRGS, Porto Alegre, RS CEP 90035-003 Brazil; 3 Hospital de Clínicas de Porto Alegre/HCPA, Porto Alegre, RS CEP 90035-903 Brazil; 4 Programa de Pós-Graduação em Saúde da Criança e do Adolescente, Faculdade de Medicina, UFRGS, Porto Alegre, RS 90035-003 Brazil

**Keywords:** B-cell acute lymphoblastic leukemia, Ectonucleotidases, Immunomodulators, Lymphocyte subpopulations

## Abstract

**Purpose:**

Although risk-stratified chemotherapy regimens improve B-cell acute lymphoblastic leukemia (B-ALL) clinical outcome, relapse occurs in a significant number of cases. The identification of new therapeutic targets as well as prognostic and diagnostic biomarkers can improve B-ALL patients' clinical outcomes. Purinergic signaling is an important pathway in cancer progression, however the expression of ectonucleotidases and their impact on immune cells in B-ALL lacks exploration. We aimed to analyze the expression of ectonucleotidases in B-ALL patients’ lymphocyte subpopulations.

**Methods:**

Peripheral blood samples from 15 patients diagnosed with B-ALL were analyzed. Flow cytometry was used to analyze cellularity, expression level of CD38, CD39, and CD73, and frequency of ${\mathrm{CD}38}^{+}{/\mathrm{CD}73}^{+}$, and ${\mathrm{CD}39}^{+}{/\mathrm{CD}73}^{+}$ in lymphocyte subpopulations. Plasma was used for cytokines (by CBA kit) and adenine nucleosides/nucleotides detection (by HPLC).

**Results:**

Comparing B-ALL patients to health donors, we observed an increase of CD4 + and CD8 + T-cells. In addition, a decrease in CD38 expression in B and Treg subpopulations and an increase in CD39^+^ CD73^+^  frequency in Breg and CD8+ T-cells. Analyzing cytokines and adenine nucleosides/nucleotides, we found a decrease in TNF, IL-1β, and ADO concentrations, together with an increase in AMP in B-ALL patients' plasma.

**Conclusion:**

As immunomodulators, the expression of ectonucleotidases might be associated with activation states, as well as the abundance of different cellular subsets. We observed a pro-tumor immunity expression profile in B-ALL patients at diagnosis, being associated with cell exhaustion and immune evasion in B-ALL.

## Introduction

Acute lymphoblastic leukemia (ALL) is the most common pediatric cancer, with B-cell ALL (B-ALL) accounting for approximately 85% of all cases. B-ALL represents one of the top leading causes of pediatric cancer death, being the peak of incidence between 2 and 5 years of age [1, 2]. Although risk-stratified chemotherapy substantially improves the clinical outcome of pediatric patients with B-ALL and results in cure rates reaching 90%, relapse still occurs in a significant number of cases [3].

In most treatment protocols, different genetic subtypes of childhood ALL are treated following risk-adapted therapy, which is tailored to the patient’s relative risk of relapse [4]. Risk of relapse evaluation is essential at diagnosis to prevent under- or over-treatment [5]. The risk stratification at diagnosis is based on a combination of age, initial white blood cell (WBC) count, early treatment response, genetic markers, and minimal residual disease (MRD) assessment. Patients are allocated into one of the following three risk groups: standard (SR), intermediate (IR), and high (HR) risk. MRD is the strongest prognostic factor, and its evaluation is important to eventually redefine the patients’ stratification risk [6, 7].

Although most patients experience substantial clinical improvement, a significant subset of children relapse, then present a poor prognosis, which urgently calls for new analytical and therapeutical approaches. Thus, identifying new targets for therapeutic interventions will enable the development of personalized therapies, increase the effectiveness of existing treatments and, subsequently, improve the clinical outcomes of B-ALL patients [8–10]. In this context, purinergic signaling has emerged in recent years as an important pathway in cancer progression, where extracellular adenosine triphosphate (ATP) and adenosine (ADO) act as signaling molecules in various biological activities and modulate the function of tumor cells, even though it still receives little attention from the field of hematological neoplasms, especially B-ALL [11–14].

In purinergic signaling, there are two different adenosinergic pathways for ADO production, the canonical and non-canonical pathways, which are modulated by several membrane surface enzymes, known as ectonucleotidases. The canonical pathway is started by Nucleoside Triphosphate Diphosphohydrolase (NTPDase1, CD39), which converts ATP to ADP and ADP to AMP, while Ecto-5’-nucleotidase (CD73) is responsible for the degradation of AMP to ADO. The non-canonical pathway is started by Nicotinamide Adenine Dinucleotide (NAD +)-Glycohydrolase (CD38), which converts NAD + to ADPR. ADPR is then converted by Ectonucleotide Pyrophosphatase/Phosphodiesterase 1 (NPP1, CD203a) to AMP, which is subsequently metabolized to ADO by CD73 [15–17].

Ectonucleotidases of the canonical pathway are expressed in several cell types: CD39 is mostly expressed in natural killer (NK) cells, monocytes, dendritic cells, B-cells, and regulatory B- and T-cells (Breg and Treg) [18], whereas CD73 is more expressed in myeloid cells, bone marrow stromal cells, thymic epithelial cells, B-cells, and Breg and Treg cells [19]. B-cells (in general, but specially Breg), Tregs, Th17 cells, NK cells, and myeloid-derived suppressor cells can co-express CD39 and CD73 [17]. CD38, ectonucleotidase of the non-canonical pathway, is expressed in lymphoid and myeloid cells, and is also expressed in red blood cells, platelets, and healthy and malignant plasma cells [20]; its expression level varies with the stage of maturation, the type of activation, and the milieu in which activation occurs [21].

The overexpression of CD38 on malignant plasma cells makes CD38 an ideal target for immunotherapy in multiple myeloma (MM) [21, 22]; and a potential target in adult acute myeloid leukemia (AML) and T-cell acute lymphoblastic leukemia (T-ALL) [23–25]. CD39 may contribute to the immunosuppression in AML [26] and MM, by impacting T cell dysfunction and exhaustion [27]. Blockade antibodies against CD73 are currently under active clinical studies for treating solid tumors (NCT03454451, NCT02503774); Furthermore, recent studies demonstrate the potential of CD73 in antileukemic immunity in AML [28]. However, little is known about the effect and function of CD38, CD39, and CD73 in B-ALL. To address this important question, we performed phenotypic and functional studies regarding the ectonucleotidases expression in the peripheral blood-derived lymphocytes of a cohort of B-ALL patients (n = 15) at diagnosis.

## Materials and methods

### Patients and controls

**Fig. 1:**
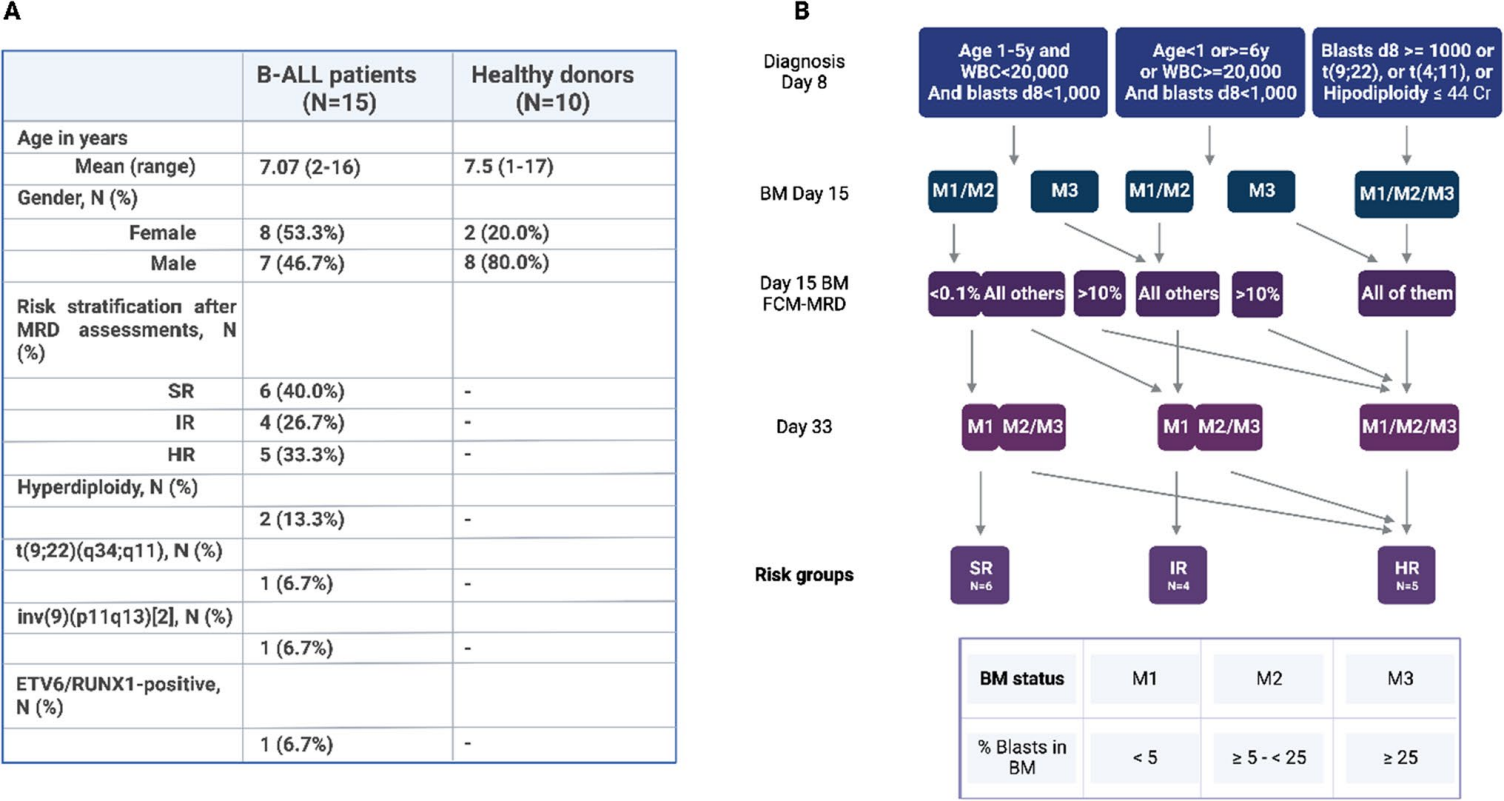
Clinical data: age in years, gender, risk stratification after MRD assessments, hyperdiploidy, t(9;22)(q34;q11) translocation, inv(9)(p11q13)[2] heteromorphism, and ETV6/RUNX-1 rearrangement of B-ALL patients and healthy donors (**a**) and schematically summarized and adapted classification of risk groups according to the ALL IC-BFM 2009 classification (**b**). B-ALL, B-cell acute lymphoblastic leukemia; BM, bone marrow; Cr, chromosomes; d8, day 8; FCM, flow-cytometry; HR, high risk; IR, intermediate risk; MRD, minimal residual disease; N, number; SR, standard risk; WBC, white blood cells; y, years

Peripheral blood samples from 15 pediatric patients diagnosed with common B-ALL were obtained from Clinical Hematology and Pediatric Oncology services from Hospital de Clínicas de Porto Alegre (HCPA), from November 2018 to April 2021. The Institutional Review Board has approved the study and informed consent was obtained from parents or legal guardians of patients and healthy donors, as according to the Declaration of Helsinki. All experiments were performed with diagnosis samples from B-ALL patients before receiving any therapy. The mean age of patients was 7.07 years (range 2–16 years), and there were seven boys and eight girls. Diagnosis of B-ALL (all patients included in the study belonged to the common subtype) was performed by antigen expression detection via flow cytometry according to EuroFlow protocols [29] (Serviço de Diagnóstico Laboratorial do HCPA), and risk stratification performed according to the ALL IC-BFM 2009 protocol (Fig. 1b). The ALL IC-BFM 2009 classification system is based on age at diagnosis, initial WBC count, early treatment response, some genetic markers and/or their molecular equivalents, aneuploidies, and MRD assessments [7]. Clinical data including genetic abnormalities of B-ALL patients are shown in Fig. 1a. Most patients carried SR (six patients) or HR (five patients), and only four patients were categorized with IR after MRD assessments on days 15, 33, and week 12 (optional). Two patients had a stratification redefinition: one of the patients was classified as IR at diagnosis and after MRD was reallocated to the HR group; and the other patient was classified as SR at diagnosis and after MRD was reallocated to the IR group. All other patients (n = 13) did not have a change in stratification after MRD assessments, remaining in the risk group defined at diagnosis. Samples of 10 healthy donors (HD), being eight boys and two girls, with mean age of 7.5 years, ranging from 1 to17 years, were obtained for controls. The study was approved by the Ethics Committees of HCPA and Universidade Federal do Rio Grande do Sul (UFRGS) (Project number: 2018–0401, CAAE number: 93973218110015327; and Project number: 2022–0094, CAAE number: 60106922000005327).

### Flow cytometric analysis and gating strategies

For surface staining, peripheral blood cells were incubated with mouse anti-human monoclonal antibodies (mAbs) for 30 min at room temperature in the dark and then lyzed and washed before analysis. The mAbs used were: CD3 APC-H7 (SK7 clone), CD4 PB (RPA-T4 clone), CD8 PE-Cy 7 (RPA-T8 clone), CD19 PE-Cy 7 (SJ25C1 clone), CD20 V450 (L27 clone), CD25 PerCp-Cy 5.5 (M-A251 clone), CD38 FITC (HIT2 clone), CD39 APC (TU66 clone), CD45 V500 (HI30 clone), and CD73 PE (AD2 clone) (all from BD Biosciences, San Diego, CA). Data acquisition was performed on a FACSCanto II flow cytometer (BD-Becton Dickinson, San Jose, CA, USA) with the standard EuroFlow instrument settings [30], and data analysis was performed using the Infinicyt® software version 2.0 (Cytognos, Salamanca, Spain).

Furthermore, we designed a tube that allowed the analysis of all main lymphocyte subpopulations in a single tube. First, we excluded debris and dead cells based on forward and side scatter (FSC/SSC), after gating leukocytes as CD45^+^ and lymphocytes on $${CD45}^{bright}$$ vs. SSC, the markers CD3 and CD19 were used to define B-cells ($${SSC}^{low}/{CD45}^{bright}/{CD3}^{-}/{CD19}^{+})$$ and T-cells $${(SSC}^{low}/{CD45}^{bright}/{CD3}^{+}/{CD19}^{-})$$. The T-cell subset was subdivided into CD4+ $${(SSC}^{low}/{CD45}^{bright}/{CD3}^{+}/{CD19}^{-}/{CD4}^{+}/{CD8}^{-})$$ or CD8 + ($${SSC}^{low}/{CD45}^{bright}/{CD3}^{+}/{CD19}^{-}/{CD4}^{-}/{CD8}^{+})$$. From CD4 + T-cells one extra population was separated, Treg cells ( $${SSC}^{low}/{CD45}^{bright}/{CD3}^{+}/{CD19}^{-}/{CD4}^{+}/{CD8}^{-}/{CD25}^{bright}$$). The B-cell subset was subdivided into Breg ($${SSC}^{low}/{CD45}^{bright}/{CD3}^{-}/{CD19}^{+}/{CD38}^{bright}$$) and other B-cells $$({SSC}^{low}/{CD45}^{bright}/{CD3}^{-}/{CD19}^{+}/{CD38}^{-/low}$$).

### High-performance liquid chromatography (HPLC) analysis

Purines levels and metabolic products of ATP hydrolysis in plasma samples were evaluated by HPLC. Firstly, samples’ proteins were denatured with 0.6 M perchloric acid and centrifuged at 4 °C, 16,000 × g for 20 min. After that, the supernatants were neutralized with 4 M KOH and centrifuged again at 4 °C, 16,000 × g for 20 min. After the second centrifugation, supernatants were collected and filtered with a 0.22 µm syringe filter (Cobetter Filtration, Hangzhou, China). Aliquots of 30 µL were applied to a reverse-phase HPLC (Shimadzu, Kyoto, Japan) using a ${C}_{18}$ column (Gemini ${C}_{18}$, 25 cm × 4.6 cm × 5 µm, Phenomenex, Torrance, CA, USA). The elution was carried out by applying a linear gradient from 100% solvent A (60 mM ${KH}_{2}{PO}_{4}$ and 5 mM of tetrabutylammonium chloride, pH 6.0) to 100% solvent B (solvent A + 30% methanol) over a 30 min period (flow rate 1.2 mL/min), as previously described by Voelter et al*.* [31]. The amount of purines was measured by absorbance at 254 nm. The retention times of standards were used as parameters for identification and quantification.

### Inflammatory markers

Plasma levels of IL-8, IL-1β, IL-6, IL-10, TNF, and IL-12 were determined by Cytometric Bead Array (CBA) according to the manufacturer’s instruction (BD Biosciences, San Jose, CA, USA) using the BD Accuri C6 flow cytometer (BD-Becton Dickinson, San Jose, CA, USA). The FCAP Array software (BD Biosciences, San Jose, CA, USA) version 3.0 was used to analyze the results and determine each cytokine concentration.

### Statistical analysis

GraphPad Prism 7 (GraphPad Software, Inc., San Diego, CA, USA) was used for statistical calculations. The Shapiro–Wilk test was performed to verify if there is normality in the distribution of samples for each variable to be analyzed. In cases of normally distributed data, parametric tests (t-test and ANOVA) were used, and when the data were not normally distributed, non-parametric tests (Mann–Whitney and Kruskal–Wallis) were used, when suitable for the experimental design in question. The adjustments for multiple testing were not performed, being a limitation of the study. For all analyses, *p* values less than 0.05 were considered statistically significant (*p < 0.05, **p < 0.01, ***p < 0.001, ****p < 0.0001).

## Results

### Analysis of lymphocyte cellularity

**Fig. 2:**
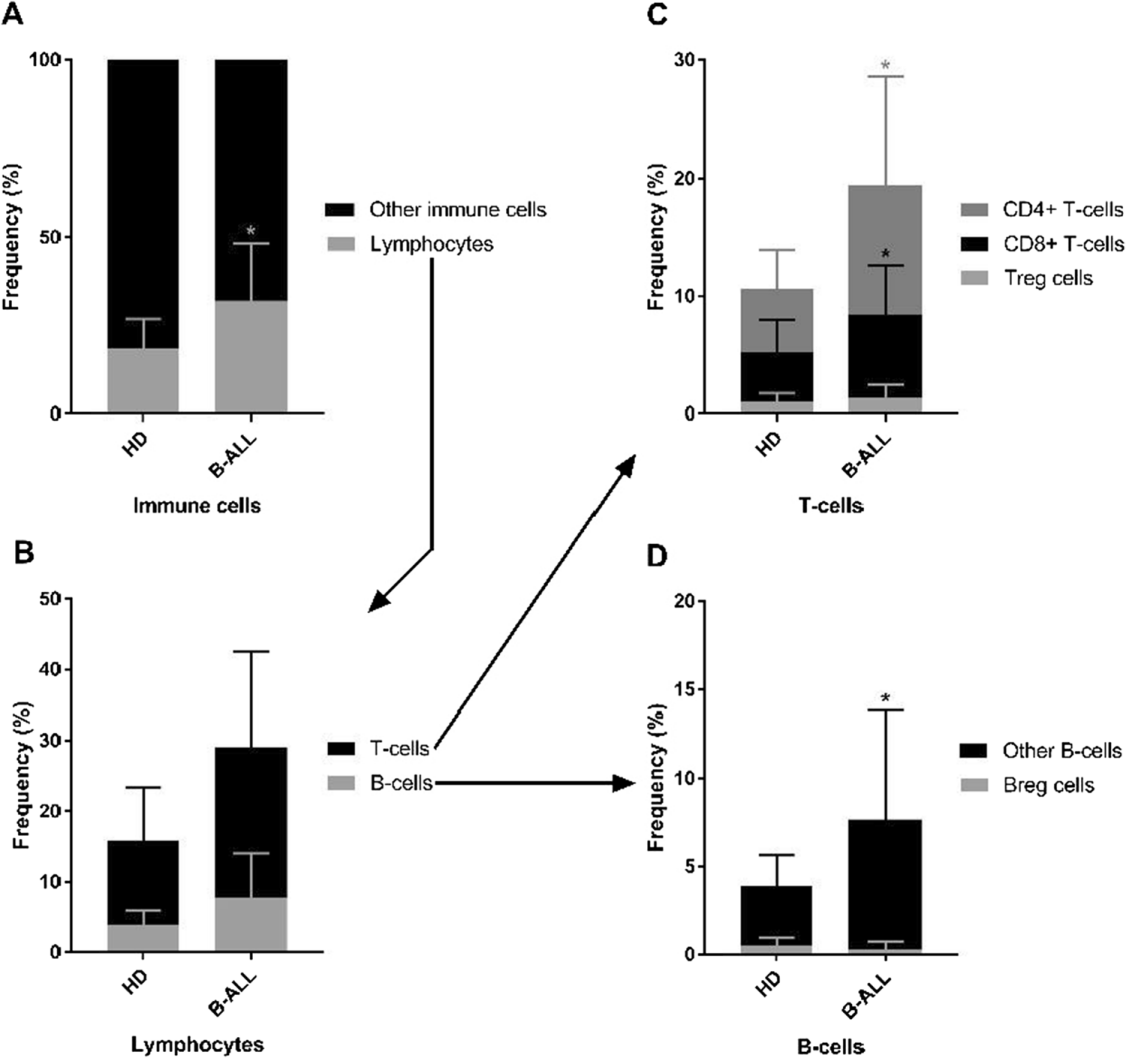
Analysis of the composition of immune cells, lymphocytes, T-cells, B-cells subsets from healthy donors (n = 10) and B-ALL patients (n = 15). Column graphs show the relative frequencies (%) of cell subsets. Within the population of immune cells, the distribution of lymphocytes and other immune cells was separated (**a**). Within the population of lymphocytes, the distribution of T-cells and B-cells was separated (**b**). Within the population of T-cells, the distribution of CD4 + T-cells, CD8 + T-cells, and Treg cells was separated (**c**). Within the population of B-cells, the distribution of Breg cells and other B-cells was separated (**d**). p-values were obtained with t-test or Mann–Whitney test. Bars represent the mean ± standard deviation. *p < 0.05. B-ALL, B-cell acute lymphoblastic leukemia; HD, healthy donors

In the B-ALL group, the analysis showed that within the population of immune cells 31.89% are lymphocytes (Fig. 2). Within the total lymphocyte population 21.39% are T-cells (approximately 11.01% CD4 + T-cells, 7.10% CD8 + T-cells, and 1.31% Treg cells), and 7.68% are B-cells (approximately 0.31% Breg cells, and 7.36% other B-cells). In the control group, within the population of immune cells 18.59% are lymphocytes. Within the total lymphocyte population 12% are T-cells (approximately 5.40% CD4 + T-cells, 4.25% CD8 + T-cells, and 0.99% Treg cells), and 3.86% are B-cells (approximately 0.49% Breg cells, and 3.36% other B-cells). A significant increase was found in B-ALL group compared to the control group for the cell frequency of the following populations: total lymphocytes (*p* = 0.0136), CD4 + T-cells (*p* = 0.0418), CD8 + T-cells (*p* = 0.0498), and in the “other B-cells” subset (*p* = 0.0264).

### The expression level of CD38, CD39, and CD73 in lymphocyte subpopulations

**Fig. 3:**
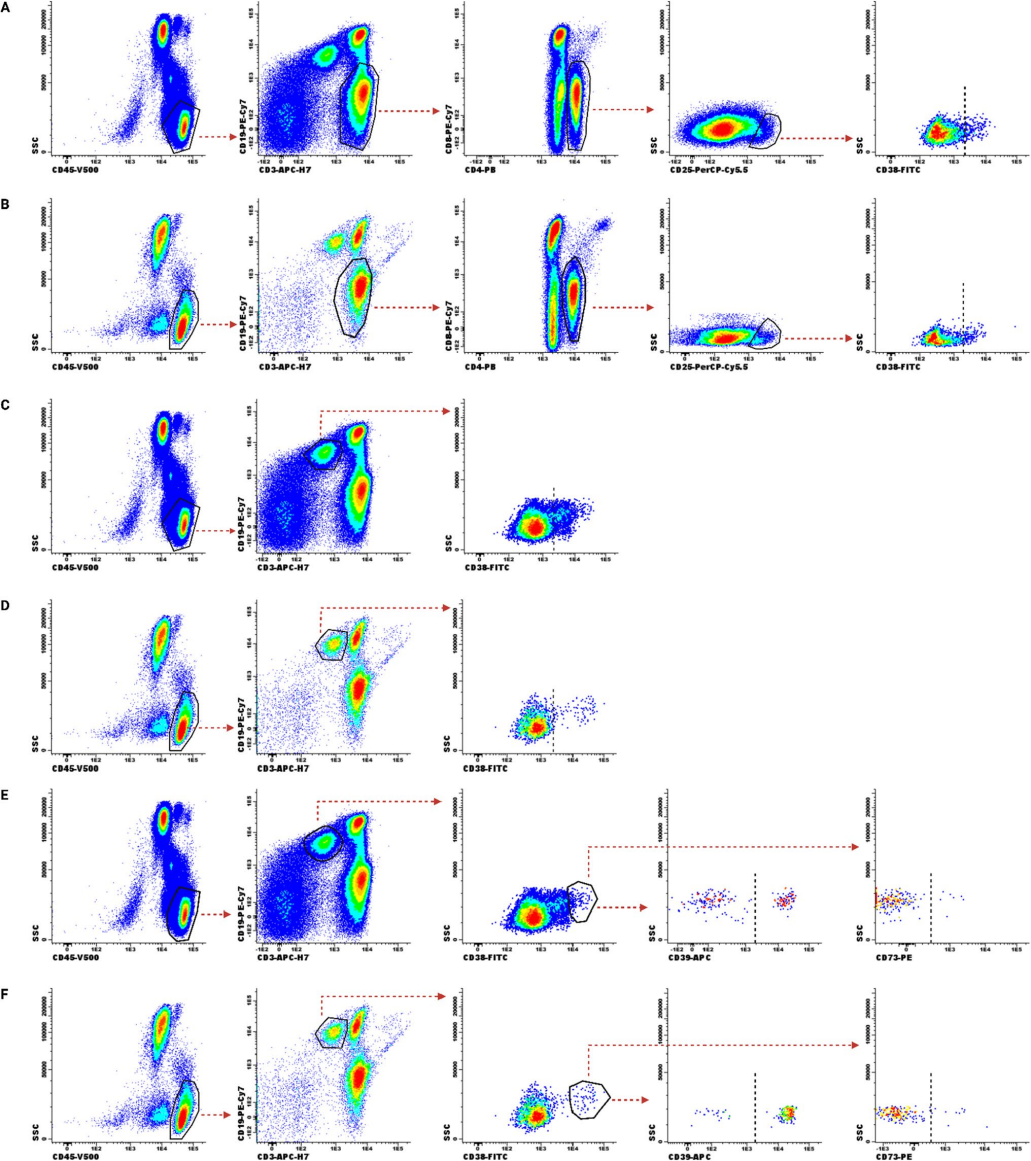
Representative flow cytometry gating strategy for the identification of Treg cells (${SSC}^{low}/{CD45}^{bright}/{CD3}^{+}/{CD19}^{-}/{CD4}^{+}/{CD8}^{-}/{CD25}^{bright};$ **a** and **b**), B-cells (${SSC}^{low}/{CD45}^{bright}/{CD3}^{-}/{CD19}^{+}$; **c** and **d**), and Breg cells (${SSC}^{low}/{CD45}^{bright}/{CD3}^{-}/{CD19}^{+}/{CD38}^{bright}$; **e** and **f**), subsets. In the Treg cells subset the last graph  represents CD38 expression of healthy donor (**a**), and of B-ALL patient (**b**). In the B-cell subset the last graph represents CD38 expression of healthy donor (**c**), and of B-ALL patient (**d**). In the Breg cells subset the last two graphs represent the CD39 and CD73 expression, respectively, from healthy donor (**e**), and B-ALL patient (**f**)

**Fig. 4:**
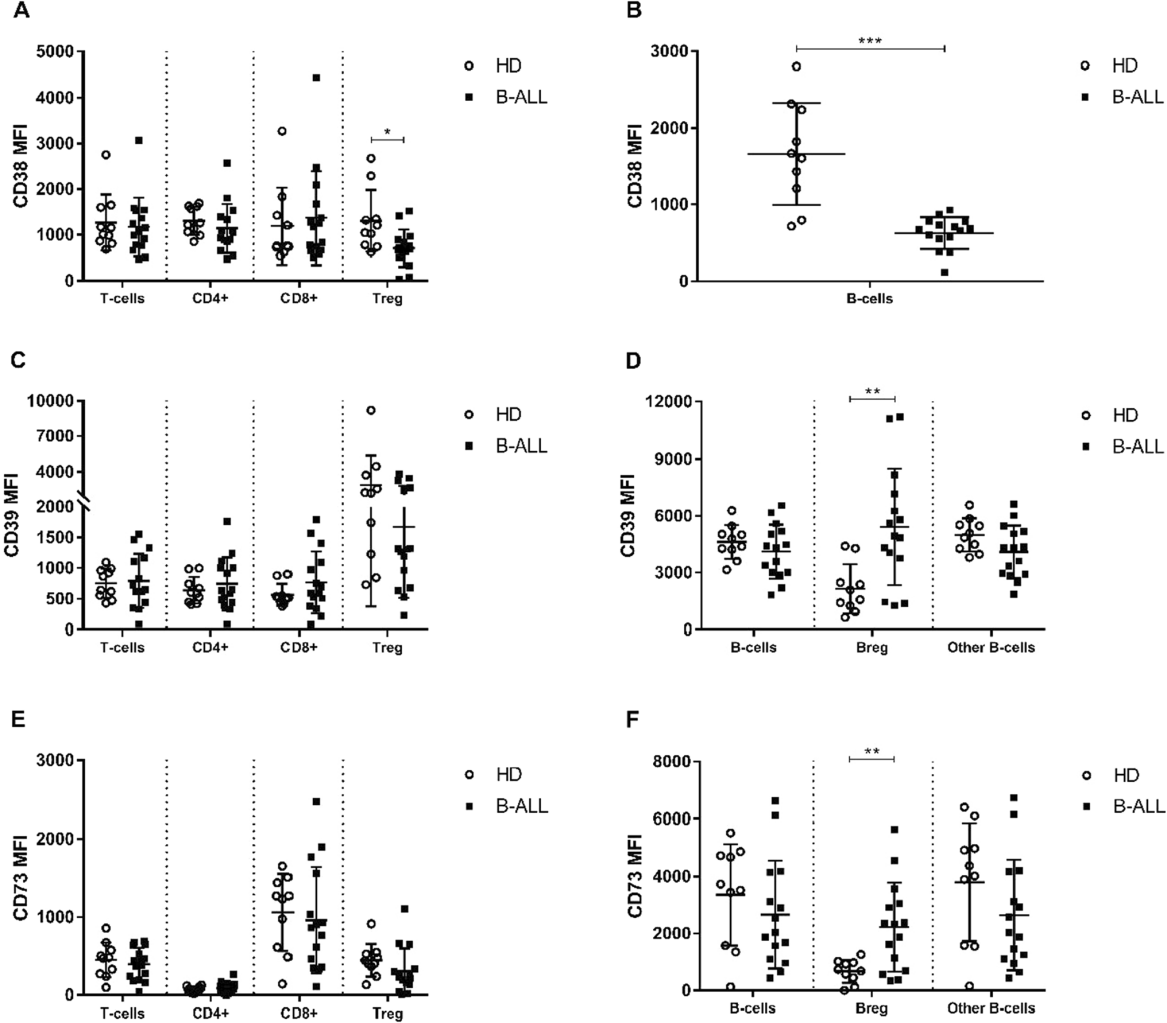
Representative summary plot of CD38 (**a** and **b**), CD39 (**c** and **d**), and CD73 (**e** and **f**) expression on T- (**a**, **c**, and **e**) and B-cells (**b**, **d**, and **f**) subpopulations from healthy donors and B-ALL patients. p-values were obtained with t-test or Mann–Whitney test. Bars represent the mean ± standard deviation. *p < 0.05, **p < 0.01, ***p < 0.001. B-ALL, B-cell acute lymphoblastic leukemia; HD, healthy donors; MFI, mean fluorescence intensity

**Fig. 5:**
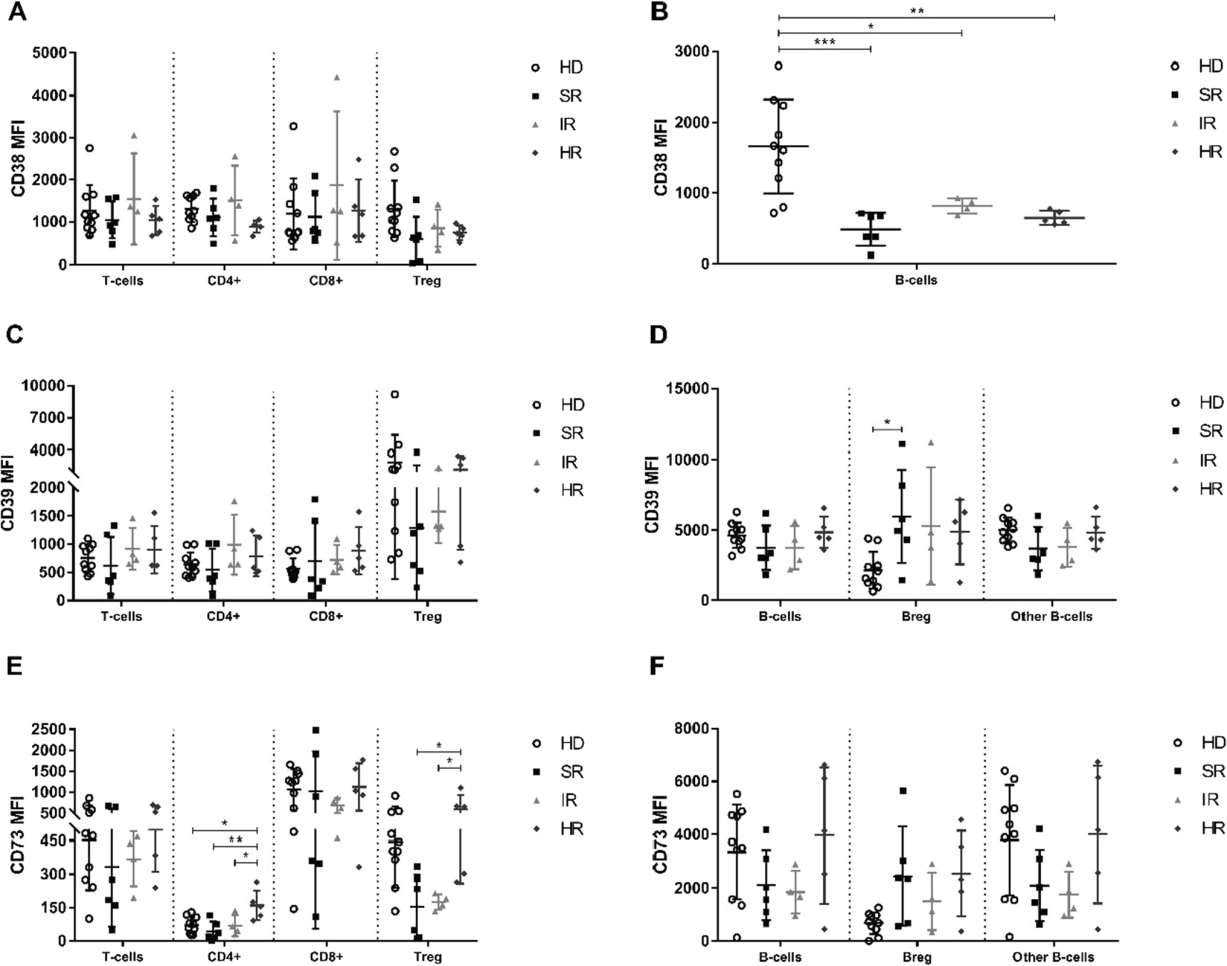
Representative summary plot of CD38 (**a** and **b**), CD39 (**c** and **d**), and CD73 (**e** and **f**) expression on T- (**a**, **c**, and **e**) and B-cells (**b**, **d**, and **f**) subpopulations from healthy donors and B-ALL patients stratified into SR, IR, and HR groups. p-values were obtained with one-way ANOVA or Kruskal–Wallis test. Bars represent the mean ± standard deviation. *p < 0.05, **p < 0.01, ***p < 0.001. B-ALL, B-cell acute lymphoblastic leukemia; HD, healthy donors; HR, high risk; IR, intermediate risk; MFI, mean fluorescence intensity; SR, standard risk

Flow cytometry gating strategy for Treg cells, B-cells, and Breg cells subsets exemplification is provided in Fig. 3. We found a significant decrease, in B-ALL group compared to the control group, of the expression level of CD38 in the subpopulations of Treg cells (*p* = 0.0137, Fig. 4a), and B-cells (*p* = 0.0007, Fig. 4b). When stratifying B-ALL patients into risk groups, we found a significant decrease in CD38 expression levels in B-cells SR (*p* = 0.0002, Fig. 5b), IR (*p* = 0.0133, Fig. 5b), and HR (*p* = 0.0015, Fig. 5b) groups when compared to control group. Furthermore, we found a significant increase, in the B-ALL group compared to the control group, of the expression level of CD39 (*p* = 0.0015, Fig. 4d) on Breg cells. By stratifying B-ALL patients into risk groups, considering CD39 expression, we only found a significant increase on Breg cells in the SR group compared to the control group (*p* = 0.0270, Fig. 5d).

We found a significant increase, in the B-ALL group compared to the control group, for the expression level of CD73 (*p* = 0.0019, Fig. 4f) on Breg cells. We found a significant increase in HR (*p* = 0.0124, Fig. 5e), SR (*p* = 0.0029, Fig. 5e), and IR (*p* = 0.0429, Fig. 5e) groups, when compared to control, for the expression level of CD73 in the population of CD4 + T-cells. We also found a significant increase in CD73 expression in the HR group compared to the SR (*p* = 0.0127, Fig. 5e), and IR (*p* = 0.0362, Fig. 5e) groups for Treg cells. A greater impact of CD73 expression on the Breg cells subpopulation was observed, especially on the HR group and specifically to the populations of CD4 + T-cells and Treg cells.

### Frequency of ${{\varvec{C}}{\varvec{D}}38}^{+}{{\varvec{C}}{\varvec{D}}73}^{+}$, and ${{\varvec{C}}{\varvec{D}}39}^{+}{{\varvec{C}}{\varvec{D}}73}^{+}$ lymphocytes

**Fig. 6:**
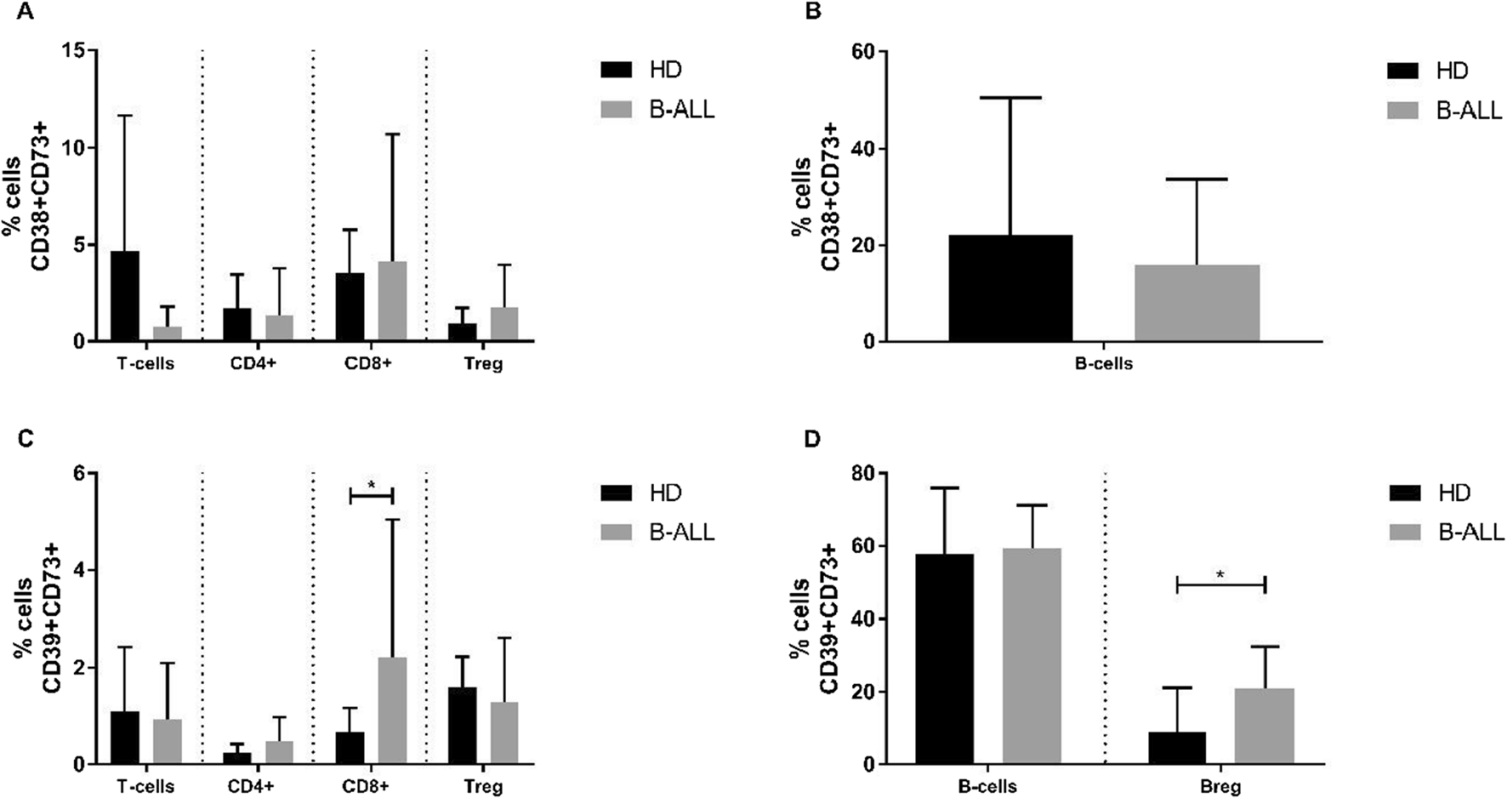
Immunophenotypic characterization for double-positive purinergic markers on T- (**a** and **c**) and B-cells (**b** and **d**) subpopulations from healthy donors and B-ALL patients. Percentage of the double-positive populations of CD38^+^ CD73^+^ (**a** and **b**) and CD39^+^ CD73^+^ (**c** and **d**) cells. p-values were obtained with t-test or Mann–Whitney test. Bars represent the mean ± standard deviation. *p < 0.05. B-ALL, B-cell acute lymphoblastic leukemia; HD, healthy donors

Concerning ${CD38}^{+}{CD73}^{+}$ double staining (Fig. 6), we did not find a significant difference in frequency when comparing B-ALL and control groups. We found a significant increase in the frequency of ${CD39}^{+}{CD73}^{+}$ cells in the B-ALL group compared to the control group in the subpopulation of CD8 + T-cells (*p* = 0.0343, Fig. 6c), and Breg cells (*p* = 0.0234, Fig. 6d).

### Concentration of cytokines and adenine nucleosides/nucleotides in plasma

**Fig. 7:**
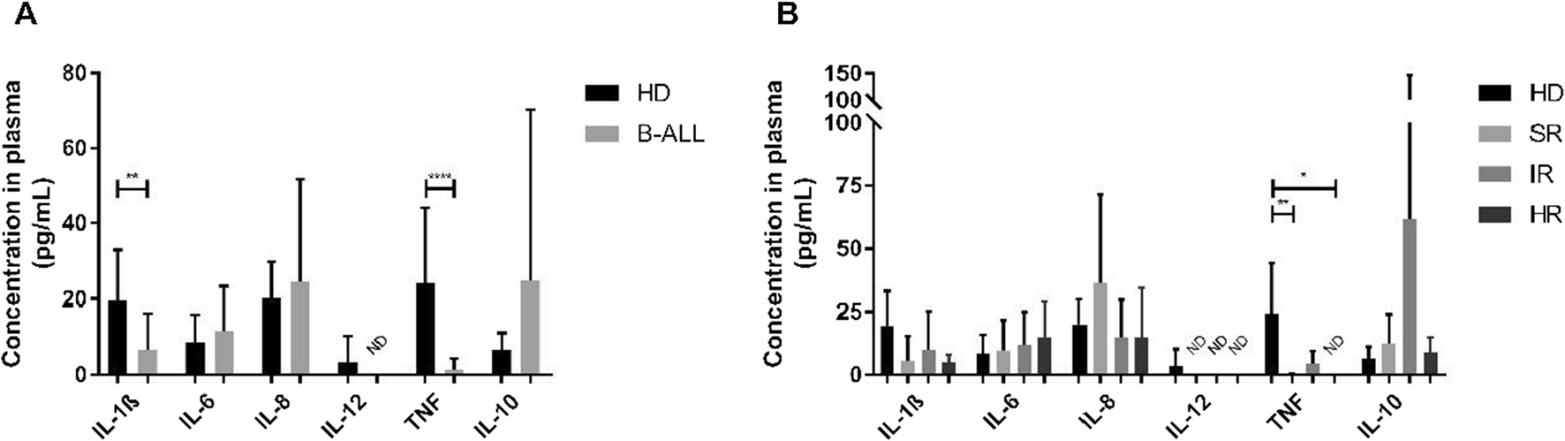
Characterization of inflammatory markers. Concentration in plasma, in pg/mL, of pro- (IL-1β, IL-6, IL-8, IL-12, and TNF) and anti-inflammatory (IL-10) cytokines in healthy donors and B-ALL patients (**a**)/healthy donors and B-ALL patients stratified into SR, IR, and HR groups (**b**). Cytokine levels were determined with the CBA kit. p-values were obtained with t-test and Mann–Whitney test (**a**), and one-way ANOVA or Kruskal–Wallis test (**b**). Bars represent the mean ± standard deviation. *p < 0.05, **p < 0.01, ****p < 0.0001. B-ALL, B-cell acute lymphoblastic leukemia; HD, healthy donors; HR, high risk; IL-1β, interleukin 1 beta; IL-6, interleukin 6; IL-8, interleukin 8; IL-12, interleukin 12; IL-10, interleukin 10; IR, intermediate risk; ND, not detected; SR, standard risk; TNF, tumor necrosis factor

We observed a significant decrease in the concentration of TNF (*p* < 0.0001, Fig. 7a), and IL-1β (*p* = 0.0090, Fig. 7a) in the B-ALL group, compared to controls. When stratifying B-ALL according to risk and comparing each with the control group, we observed a significant decrease in the concentration of TNF in the SR (*p* = 0.0088, Fig. 7b) and HR (*p* = 0.0227, Fig. 7b) groups.

**Fig. 8:**
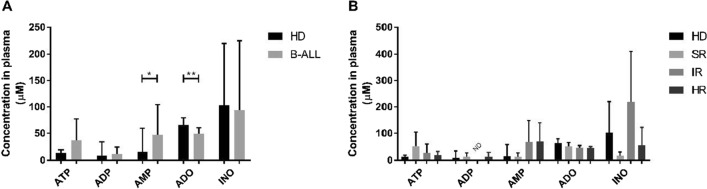
Characterization of purines levels and metabolic products of ATP hydrolysis. Concentration in plasma, in µM, of extracellular adenine and adenosine nucleotides (ATP, ADP, AMP, ADO, and INO) in healthy donors and B-ALL patients (**a**)/healthy donors and B-ALL patients stratified into SR, IR, and HR groups (**b**). Purine levels and metabolic products of ATP hydrolysis were determined by HPLC. p-values were obtained with t-test or Mann–Whitney test (**a**), and one-way ANOVA or Kruskal–Wallis test (**b**). Bars represent the mean ± standard deviation. *p < 0.05, **p < 0.01. ADO, adenosine; ADP, adenosine diphosphate; AMP, adenosine monophosphate; ATP, adenosine triphosphate; B-ALL, B-cell acute lymphoblastic leukemia; HD, healthy donors; HR, high risk; INO, inosine; IR, intermediate risk; ND, not detected; SR, standard risk

Lastly, we only observed a significant increase in the concentration of AMP (*p* = 0.0316, Fig. 8a) and a significant decrease in the concentration of ADO (*p* = 0.0074, Fig. 8a) in the B-ALL group, compared to control group.

## Discussion

Malignancy is not merely a cluster of neoplastic cells but a microenvironment that contains endothelial cells, fibroblasts, structural components, and immune cell infiltrates that affect cancer development, invasion, and metastasis [32]. Many recent studies have shed a light on this complex interaction between tumor-derived immune cells, modulated not to attack neoplastic cells, and non-tumor-derived immune cells, responsible for attacking neoplastic cells in solid tumors [33, 34]. These studies stated that high values of infiltrating lymphocytes in the tumor microenvironment can predict treatment outcomes and can be a potential prognostic indicator [35, 36]. Therefore, phenotypic changes in the B-ALL microenvironment (bone marrow) can be transmitted to peripheral blood and aid in diagnosis, prognosis, and development of new therapies for patients with B-ALL. One of the objectives of the present study was to characterize the immune system of patients with B-ALL highlighting the clinical relevance of total lymphocytes and their subpopulations in peripheral blood through the lenses of purinergic signaling, an important system for cancer immunosuppression and pro-tumor immunity.

We observed a significant increase in CD4 + T-cells and CD8 + T-cells populations in the B-ALL group. Previous studies have addressed the question of absolute number and function of T-cells in peripheral blood after chemotherapy in patients with acute leukemias [37, 38], as well as reported a decrease in the absolute number of T-cells in patients with ALL [39]. Le Dieu et al. characterized T-cells in peripheral blood from newly diagnosed AML patients compared to age-matched healthy donors and found that the absolute number of T-cells is increased with particular amplification of the CD8 + T-cell subset; interestingly, Le Dieu et al. evaluated the gene expression profile of these T-cells and detected aberrant patterns of activation. As already observed in chronic lymphocytic leukemia (CLL) and more recently in AML, a T-cell dysfunction has been identified which may contribute to the failure of the host’s immune response against leukemic blasts [40]. Similarly, Bar et al. demonstrated an association between high absolute lymphocyte counts at diagnosis and worse outcomes in AML [41]. Therefore, although B-ALL patients have a higher number of T-cells, this increase may be associated with cellular exhaustion phenotypes and/or immune evasion.

Despite being part of a physiological aid to prevent excessive inflammation and tissue damage, in tumor biology ectonucleotidases can contribute to generating immunosuppressive conditions through the production of adenosine [42]. Similar to other mechanisms that are hijacked and serve to pro-tumor purposes, tumor cells can benefit from the expression of ectonucleotidases, mainly CD38, CD39, and CD73. In cancer, including hematological malignancies such as B-ALL, overexpression of ectonucleotidases, mainly CD39 and CD73, has been associated with increased cell resistance and proliferation as well as modulation of immune responses into tolerance [43].

However, regarding the expression of CD38, as lesser known part of the purinergic system, we observed CD38 expression was significantly reduced in subpopulations of B-cells and Treg cells in the B-ALL compared to HD, regardless of the risk group (SR, IR or HR). CD38 expression on hematopoietic cells is variable, being a temporal characteristic of the activation state and/or stage of cells’ maturation, being quite high in activated T and B lymphocytes. CD38 is involved in lymphocyte activation and homing, in addition to performing several other functions including adhesion to the endothelium. Nevertheless, its role in B-cells is not yet clearly defined [44, 45]. Zupo et al*.* have shown that peripheral CLL-derived B-cells expressed CD38 in abundance while expression was low to absent in another group of CLL patients. Moreover, CD38 expression identified a subset of B-cells with defined properties, including the propensity to undergo apoptosis and a rapid intracellular calcium mobilization after cross-linking of surface Ig (sIg); while low to absent expression of CD38 identified another subset of B-cells with defined properties including resistance to undergo apoptosis and unresponsive to stimulation with anti-IgM antibodies. These observations raise a number of issues regarding the role of CD38 in B-cell activation, calcium mobilization, and apoptosis [46]. The level of CD38 expression is known to correlate the suppressor function of Treg cells [47], where low expression may define a Treg subset with reduced immunosuppressive capacity [48–50]. Thus, it can be inferred that the presentation of lower CD38 expression in the patients analyzed in our B-ALL cohort might be associated with lower activation features on B-cells and lower immunosuppressive capacity of Treg cells, or greater cell maturation, although better understanding of these intricacies is still needed.

Regarding CD39 and CD73, we observed a significant increase in the B-ALL group of the expression level of CD39 and CD73, as well as in the frequency of ${CD39}^{+}{CD73}^{+}$ double positive Breg cells. When patients were classified according to risk groups we observed a significant increase, in the SR group, of CD39 expression levels in the Breg subpopulation. It has already been observed that Breg cells are able to suppress effector T-cells by the production of ADO and IL-10. In fact, the production of ADO, by the overexpression of CD39 and CD73 in Breg cells, not only triggers the inhibition of T-cells but also increases the frequency of Breg cells themselves [51]. Furthermore, Kaku et al*.* demonstrated a regulatory role for CD39 and CD73-expressing B cells, on experimental colitis, through ADO generation in an IL-10-independent manner [52]. In the CD8 + T-cells population, we also observed a significant increase in the frequency of cells expressing both ${CD39}^{+}$ ${CD73}^{+}$ in the B-ALL group. A recent study highlights that the co-expression of CD39 and CD103 (which is involved in adhesion and activation) identifies a subset of tumor-reactive CD8 + T-cells in solid human tumors [53]. Furthermore, evidence shows that the tumor microenvironment drives function exhaustion in a subset of CD8 + T-cells, conditioning cell surface expression of CD39, an immunosuppressive molecule that can be therapeutically targeted to restore the effector T-cell function [54]. These Breg and CD8 + T-cells could be producing more ADO through the canonical pathway of the purinergic system, since CD39 overexpression converts more ATP into AMP, a substrate of CD73 ectonucleotidase for the production of ADO. Greater ADO production leads to greater immunosuppression and immune evasion, which can lead to B-ALL progression. In addition, the expression of CD39 on CD8 + T-cells in the peripheral blood of B-ALL patients might be a mark of exhaustion and reactivity, as this behavior has already been observed in solid tumors [55].

Inflammation is an immune response to damage and is mediated mainly by cytokines, which play important roles in modulating cell survival, proliferation, differentiation, and immune response [56, 57]. Cytokines are classified as pro-inflammatory (e.g. IL-1β, IL-6, IL-8, IL-12, and TNF), and anti-inflammatory (e.g. IL-10, and TGF-β) [58, 59]. In our analysis of inflammatory markers in the peripheral blood of patients with B-ALL we found a statistically significant decrease in the concentration of TNF and IL-1β. TNF is produced by normal B-cells and it functions as an autocrine growth and differentiation factor for these cells [60–62]. In some malignancies, such as B-CLL and hairy cell leukemia, it has been suggested that this cytokine may represent an autocrine growth factor for these neoplasms [63, 64]. TNF inhibits the growth of normal hematopoietic progenitor cells but paradoxically increases the proliferation of neoplastic cells in patients with myeloproliferative neoplasms, myelodysplastic syndrome, and Fanconi anemia [65–67]. IL-1 is a master regulator of inflammation, innate immunity, and hematopoiesis [24, 68]. We believe that a lower concentration of TNF in peripheral blood may be associated with higher proliferation of hematopoietic progenitor cells but lower proliferation of differentiated B-cells, similarly a lower concentration of IL-1β may define a microenvironment less prone to cell survival and cancer progression in B-ALL. The author’s hypothesis is that even with an increase in some populations of immune cells in B-ALL we are observing a decrease in pro-inflammatory cytokines, mainly TNF and IL-1β, due to immune exhaustion. Even with high levels of circulating lymphocytes in peripheral blood these cells are likely to not be able to perform their functions efficiently such as, for example, secretion of pro-inflammatory cytokines.

Another important factor that has a significant impact on the course of cancer is the biochemical composition of the tumor microenvironment and, eventually, the peripheral blood, in the case of hematologic malignancies [69, 70]. It has already been reported that purine nucleotides and nucleosides are significant components of the bone marrow microenvironment in AML [71]. Adenine nucleotides (ATP) are released into the extracellular space by necrotic and inflammatory cells, or tumor cells [72]. During neoplastic growth, adenosine and adenine nucleotides, abundant components of the tumor microenvironment, are potent modulators of the immune response, such as, in the control of cytokine release and in immune system-cancer interactions [73, 74]. Our analysis of extracellular adenine nucleosides/nucleotides in the peripheral blood of B-ALL patients found a statistically significant increase in AMP concentration and a decrease in ADO concentration. It is known that AMP can be used as a ligand to activate adenosine receptors, mainly A1 receptor [74, 75]. Cellular uptake of adenosine may also be occurring, decreasing its concentration in the extracellular space and consequently minimizing its effects [76]. Opportunistic uptake of extracellular nutrients through multiple endocytic mechanisms has been described as a primary feature of cancer metabolism [77, 78]. For example, adenosine uptake leads to ATP boosting in head-neck cancer cells [79]. We observed a difference in the expression of CD39 and CD73 ectonucleotidases and an alteration in AMP and ADO metabolites; Therefore, we can suggest that a function rearrangement may be taking place in the canonical ADO production pathway of the purinergic system in B-ALL.

It is the expression of various immune mediators and modulators, as well as the abundance and activation state of different cell types that dictate in which direction the balance is tilted and whether inflammation promotes tumor growth or antitumor immunity [80, 81]. In established neoplasms, this balance is profoundly tilted towards pro-tumor immunity, even more at the time of diagnosis in patients where no therapeutic intervention has been done yet [82]. Even though, no single immune modulator can faithfully reproduce the complexity of immunity in cancer, which makes it difficult to assess the overall impact of immunity and inflammation on tumorigenic events, a select combination of mediators might be involved in key events leading to tumor progression and immune evasion. However, it is clear that neoplasm-promoting immune modulation and antitumor immunity coexist at different points along the path of neoplastic development and progression [83, 84], including in the context of B-ALL. In this sense, further studies are needed to provide information on purinergic characteristics in B-ALL patients to optimize patient risk allocation as well as the development of new antileukemic agents. Here we highlight the potential role of purinergic signaling in various immune cell subsets and evaluate the immune profile of B-ALL patients at the moment of diagnosis compared to healthy donors and stratify the patients according to risk allocation.

## Data Availability

All data and material are available upon reasonable request.
